# Humoral immune response following a third SARS-CoV-2 mRNA vaccine dose in solid organ transplant recipients compared with matched controls

**DOI:** 10.3389/fimmu.2022.1039245

**Published:** 2022-12-09

**Authors:** Daniel Balsby, Anna Christine Nilsson, Inge Petersen, Susan O. Lindvig, Jesper Rømhild Davidsen, Rozeta Abazi, Mikael K. Poulsen, Inge K. Holden, Ulrik S. Justesen, Claus Bistrup, Isik Somuncu Johansen

**Affiliations:** ^1^ Department of Infectious Diseases, Odense University Hospital, Odense, Denmark; ^2^ Department of Clinical Research, University of Southern Denmark, Odense, Denmark; ^3^ Department of Clinical Immunology, Odense University Hospital, Odense, Denmark; ^4^ Open Patient data Explorative Network, Odense University Hospital, Odense, Denmark; ^5^ Department of Respiratory Medicine, Odense University Hospital, Odense, Denmark; ^6^ Department of Gastroenterology, Odense University Hospital, Odense, Denmark; ^7^ Department of Cardiology, Odense University Hospital, Odense, Denmark; ^8^ Department of Clinical Microbiology, Odense University Hospital, Odense, Denmark; ^9^ Department of Nephrology, Odense University Hospital, Odense, Denmark

**Keywords:** Solid organ transplant, humoral response, COVID-19 vaccine, comorbidities, third dose

## Abstract

**Background:**

Solid organ transplant (SOT) recipients have shown suboptimal antibody response following COVID-19 vaccination. Several risk factors for the diminished response have been identified including immunosuppression and older age, but the influence of different comorbidities is not fully elucidated.

**Method:**

This case-control study consisted of 420 Danish adult SOT recipients and 840 sex- and age-matched controls, all vaccinated with a third homologous dose of either BNT162b2 (Pfizer–BioNTech) or mRNA-1273 (Moderna) vaccine. The primary outcome was differences in humoral immune response. The secondary outcome was breakthrough infections. Additionally, we looked for factors that could predict possible differences between the two groups.

**Results:**

Response rate increased from 186/382 (49%) to 275/358 (77%) in SOT recipients and remained on 781/790 (99%) to 601/609 (99%) in controls following a third vaccine dose. SOT recipients had significantly lower median antibody concentrations after third dose compared to controls (332.6 BAU/ml vs 46,470.0 BAU/ml, p <0.001). Lowest median antibody concentrations were seen in SOT recipients with liver disease (10.3 BAU/ml, IQR 7.1-319) and diabetes (275.3 BAU/ml, IQR 7.3-957.4). Breakthrough infections occurred similarly frequent, 150 (40%) among cases and 301 (39%) among controls (p = 0.80).

**Conclusion:**

A third COVID-19 vaccine dose resulted in a significant increase in humoral immunogenicity in SOT recipients and maintained high response rate in controls. Furthermore, SOT recipients were less likely to produce antibodies with overall lower antibody concentrations and humoral immunity was highly influenced by the presence of liver disease and diabetes. The prevalence of breakthrough infections was similar in the two groups.

## Introduction

Coronavirus disease 2019 (COVID-19) caused by the severe acute respiratory syndrome coronavirus 2 (SARS-CoV-2), has since its outbreak in late 2019 affected individuals and healthcare systems worldwide. Solid organ transplant (SOT) recipients have lower antibody concentrations and a lower proportion of responders following COVID-19 vaccination compared to the general population (1–4).

In a meta-analysis based on 82 prospective observational studies, SOT recipients were shown to be 16 times less likely to seroconvert after one dose compared with immunocompetent controls (5). They also found SOT recipients to be the least responsive group of immunocompromised patients when patients with immune mediated inflammatory disorders, haematological cancers, and solid cancers were included. Our group and others have previously found that after a three-dose regimen of COVID-19 vaccination, the proportion of responders among SOT recipients has been ranging from 49-80% thus leaving many without a response (6–11). This information is compatible with previous findings of diminished response to other vaccines (12, 13). This diminished response among SOT recipients to COVID-19 vaccination is associated with several host-related factors such as the degree of immunosuppressive treatment, increasing age, and time since transplantation (14). However, the impact of comorbidities is yet to be clarified and would be of great importance to identify patients with low vaccination responses. Following vaccination, the half-life of SARS-CoV-2 specific antibodies among SOT recipients is unclear. An improved understanding of this would provide important information about the re-vaccination strategies for optimal immunogenicity (15, 16).

In this case-control study, we studied differences in the humoral immune response among SOT recipients and age- and sex-matched non-SOT controls following a three-dose regimen of an mRNA-based SARS CoV-2 vaccination. Furthermore, we explored potential differences in breakthrough infections, and finally, we investigated factors that could predict possible differences in antibody responses between the two groups.

## Method and materials

### Study design and participants

In our study, 420 Danish adult SOT recipients were recruited from the COVAC-Tx study (Danish Ethical Committee, record no. 77786) (11). Since January 29, 2021, all SOT recipients (≥18 years of age) followed at Odense University Hospital in Region of Southern Denmark were invited to participate in the COVAC-Tx study. The SOT study population has previously been described (11). Controls were identified through the National Cohort Study of Effectiveness and Safety of SARS-CoV-2 vaccines (ENFORCE) study, an open-label, nonrandomized, parallel group, phase 4 study that enrolled Danish citizens before their first COVID-19 vaccination (clinicaltrials.gov identifier: NCT04760132) (17). Case participants and controls were matched in a 1:2 ratio according to sex and 5-year age groups. All participants provided written and oral consent before inclusion, and all received vaccination as part of the national COVID-19 vaccination program. Participants treated with monoclonal antibodies before the scheduled samples were excluded from further analyses.

The COVAC-Tx study was conducted in accordance with the Declaration of Helsinki and approved by the Regional Committees on Health Research Ethics for Southern Denmark January 29, 2021 (protocol code S-20210007C) and the amendment approved February 9, 2022. The ENFORCE study was approved by the Danish Medicines Agency (Eudra CT number:2020-006003-42) and by the Ethics Committee of the Central Denmark Region (#1-10-72-337-20).

### Data

All Danish residents are assigned a unique personal identification number (CPR) permitting data linkage of individual records between different governmental registries. Data regarding vaccination date and type was achieved through the Danish Vaccination Registry while comorbidities and hospital admissions were collected through the Danish National Patient Register. Lists of medication for cases were retrieved from the patient hospital records. SARS-CoV-2 PCR test results were obtained through the Danish national microbiology database MiBa (Statens Serum Institut, Copenhagen, Denmark). All data was entered into electronic clinical records using Research Electronic Data Capture REDCap which is hosted by OPEN (Open Patient data Explorative Network).

### Comorbidities

Comorbidities were identified based on the Quan’s coding algorithms for ICD-10 codes in years 2016-2020 (18). When relevant comorbidities were combined as follows: Diabetes with or without chronic complication; cancer: any malignancy combined with metastatic solid tumours. In the following categories relevant SOT recipients were excluded: heart disease (myocardial infarction and/or congestive heart failure), chronic pulmonary disease; liver disease (mild and/or moderate/severe liver disease) and renal disease. This prevented the inclusion of SOT’s as comorbidity.

### Blood sampling

For cases and controls, blood samples were scheduled before (0-14 days) the participants’ first SARS-CoV-2 vaccination and 90 days ( ± 14 days), 180 days ( ± 14 days) and 365 days ( ± 14 days) after first vaccination. Furthermore, blood samples were planned before (0-5 days) the second and third dose as well as 28 days ( ± 14 days) after. Antibody concentrations were measured at each visit. Immunogenicity of COVID-19 vaccination was investigated by measuring the humoral immunity against SARS-CoV-2 through analysis of IgG-antibodies targeted against the receptor binding domain (RBD) in the S1-subunit of the spike protein in SARS-CoV-2 as published by Balsby et al (11). RBD is an important target-antigen for virus neutralizing antibodies. For cases, 5 ml whole blood was collected at each visit and 150 µl plasma was used to measure the SARS-CoV-2 spike S1 IgG response using the SARS-CoV-2 IgG II Quant Assay (Abbott Laboratories). The relationship between the Abbott arbitrary units (AU)/mL unit and the World Health Organization international standard for anti-SARS-CoV-2 immunoglobulin BAU/mL unit follows the equation BAU/mL = 0.142 × AU/mL (as established by the manufacturer), corresponding to a cut-off of 7.1 BAU/mL (19). Measuring range for this assay is 7.1 - 5680 BAU/mL. Results >5680 BAU/mL are reported as such. For controls, 6 ml whole blood was collected at each visit and 150 µl plasma was used to measure levels of SARS-CoV-2 spike and receptor-binding domain (RBD) antibodies using a diagnostic multiantigen serology assay (Meso Scale Diagnostics LLC, Rockville, MD) at the Department of Infectious Diseases, Aarhus University Hospital. The relationship between the Meso Scale arbitrary units (AU)/mL and the BAU/mL unit follows the equation BAU/mL = 0.0272 × AU/mL. The manufacture did not provide a positive cut-off value for Meso Scale, however, following the internal validation, a cut-off of 27.2 BAU/mL for a positive response was chosen.

### Outcomes

The primary outcome of this study was humoral immune response following SARS CoV-2 vaccination among SOT recipients and matched controls. Secondary outcome was breakthrough infections, and additionally, we examined potential factors that could predict possible differences between the two groups.

### Statistical analysis

Categorical data are described as numbers. Group comparisons were performed using the Chi-square test. Characteristics of continuous variables are reported as medians with interquartile ranges (IQRs) and compared using non-parametric test of equal medians. Trends of mean log-scale antibody levels (with 95%CI) were estimated separately for controls and transplant groups. Changes in antibody concentrations between time points were estimated using linear mixed-effects regression models and reported as mean differences with 95% confidence intervals (CI). Due to violations of the assumption of normally distributed residuals, a bootstrapping procedure with 1000 repetitions was used in the estimation process. Furthermore, as the residuals in the SOT group demonstrated significantly higher variance after third vaccine, the model allowed for heteroscedasticity. Statistical significance was set at P < 0.05 Stata 17 was used for the analyses (StataCorp. 2021. Stata Statistical Software: Release 17. College Station, TX: StataCorp LLC).

## Results

### Characteristics

**Figure 1:**
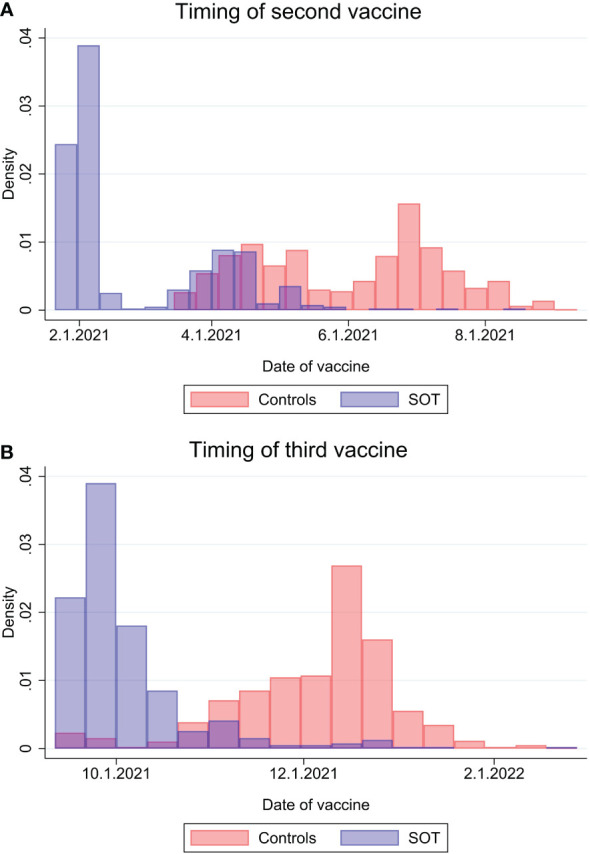
Timing of second SARS-CoV-2 vaccine dose **(A)** and third vaccine dose **(B)** among solid organ transplant recipients and matched controls.

The general characteristics of the study population are summarized in **Table 1**. Timing of SARS-CoV-2 vaccinations among SOT recipients and matched controls are shown in **Figure 1**, which illustrates how the SOT recipients were vaccinated earlier than the general population. The median age of the case and control group was 57.2 years (IQR 47.7-66.3) and 60 (IQR 51-69), respectively, with 60.2% males in both groups. Diabetes mellitus (n=86, 20.5%) cancer (both hematological and solid organ) (n=28, 6.7% and peripheral vascular diseases (n=25, 6.0%) were the three most frequent comorbidities in SOT recipients. Among controls, cancer (6.8%), diabetes mellitus (5.5%) and chronic pulmonary disease (4.5%) were the most frequent. All cases received the BNT162b2 vaccine (Pfizer, BioNTech) whereas 52% of controls received the BNT162b2 vaccine and 48% the mRNA-1273 vaccine (Moderna). Of the 420 cases and 840 controls included in this study, serum after third dose was obtained from 358 (85%) cases and 609 (73%) controls. The median time from second to third dose for cases and controls was 229 days (IQR 191-241) and 185 days (IQR 168-208), p = <0.001. Median time from third dose to blood sampling was 41.5 days (IQR 33-58) vs 29 (IQR 27-34), p ≤0.001, cases and controls respectively (**Table 2**).

**Table 1:** Baseline characteristics of solid organ transplant recipients and matched controls after third dose of a SARS-CoV-2 mRNA vaccine.

Baseline characteristics	SOT recipients (n=420)Median (IQR) or n (%)	Controls (n=840)Median (IQR) or n (%)
**Demographics**		
Age	57.2 (47.7-66.3)	60 (51-69)
Sex (males)	253 (60.2)	506 (60.2)
**Comorbidities**		
Organ transplantation	420 (100)	–
Diabetes Mellitus	86 (20.5)	46 (5.5)
Cancer	28 (6.7)	57 (6.8)
Peripheral vascular disease	25 (6.0)	8 (1)
Chronic pulmonary disease^b^	20 (4.8)	38 (4.5)
Heart disease^a^	19 (4.5)	30 (3.6)
Renal disease^d^	17 (4.1)	6 (0.7)
Cerebrovascular disease	15 (3.6)	20 (2.4)
Rheumatic disease	12 (2.9)	12 (1.4)
Liver disease^c^	11 (2.6)	13 (1.6)
**Type of transplant**		
Kidney Liver	311 (74.1) 68 (16.2)	–
Heart	18 (4.3)	–
Lung	16 (3.8)	–
Combined	7 (1.7)	–
**Immunosuppressive treatment**		
Prednisolone	97 (23)	Lacking data
CNI^e^	331 (79)	–
Proliferation inhibitor^f^	388 (92)	–

^a^Heart transplants excluded. ^b^Lung transplants excluded. ^c^Liver transplants excluded. ^d^Kidney transplants excluded ^e^Calcineurin inhibitor (CNI): tacrolimus and cyclosporine. ^f^Proliferation inhibitors: mycophenolate and azathioprine.

**Table 2:** Immunogenicity among solid organ transplant recipients and matched controls following a third dose of a SARS-CoV-2 mRNA vaccine.

Characteristics	SOT recipients (n=382)Median (IQR) or n (%)	Controls (n=790)Median (IQR) or n (%)	*p*-value
**Vaccine received**			
BTN162b2	382 (100)	411 (52)	<0.001
mRNA-1273	0 (0)	379 (48)	
**Timing**			
Time between second and third dose (days)	229 (189-241)	185 (168-208)	<0.001
Time between third dose and blood sampling (days)	41.5 (33-58)	29 (27-34)	<0.001
**Immunogenicity**			
Responders after second dose	186/382 (49)	781/790 (99)	<0.001
Responders after third dose	275/358 (77)	601/609 (99)	<0.001
Antibody concentration after second dose	≤7.1(≤7.1-73.2)	12742 (4683-28772)	<0.001
Antibody concentration after third dose	332.6 (11.9-1349.4)	46470 (31694-50374)	<0.001
Seroconversion**^a^**	100/182 (55)	1/8 (12.5)	0.02
Waned immunity**^b^**	1/176 (0.6)	1/601 (0.2)	0.40
**Comorbidities and antibody concentrations**			
Diabetes (n=79)	275.3 (7.3-957.4)	44835.0 (31733.8-50622.7)	
Cancer (n=26)	334.3 (7.3-791.2)	39709.6 (17122.2-48341.4)	
Peripheral vascular disease (n=21)	313.8 (110.4-574.3)	47070.8 (43422.2-51149.7)	
Chronic pulmonary disease^d^ (n=17)	431.3 (208.9-1054.3)	44232.3 (12269.5-49090.3)	
Heart^c^ (n=14)	345.6 (26.7-1543.6)	46961.0 (33147.2-50056.7)	
Cerebrovascular disease (n=14)	325.3 (132.7-1244.7)	49097.8 (29547.9-50373.9)	
Renal disease^f^ (n=13)	1149 (≤7.1-1863.0)	43422.2 (9128.1-50907.3)	
Rheumatic disease (n=12)	435.3 (8.7-985.0)	47918.9 (40956.5-50589.8)	
Liver disease^e^ (n=9)	10.3 (≤7.1- 319.2)	44794.3 (24178.7-49841.4)	
**Type of organ transplanted**			
Kidney (n=264)	272.2 (7.3-1224.4)	–	
Liver (n=61)	665.0 (137.9-1862.9)	–	
Heart (n=16)	797.5 (14.7-1256.0)	–	
Lung (n=11)	≤7.1 (≤7.1-486.6)	–	
Combined (n=6)	375.1 (≤7.1-791.2)	–	
**Immunosuppressive treatment**			
Prednisolone	147.6 (≤7.1-1201.6)	Lacking data	
CNI^g^	329.4 (10.3-1263.8)	–	
Proliferation inhibitor^h^	313.8 (9.6-1349.4)	–	

^a^Seronegative patients becoming seropositive after third dose. ^b^Participants with waned immunogenicity after third dose. ^c^Heart transplants excluded. ^d^Lung transplants excluded. ^e^Liver transplants excluded. ^f^Kidney transplants excluded. ^g^Calcineurin inhibitor (CNI): tacrolimus and cyclosporine. ^h^Proliferation inhibitors: mycophenolate and azathioprine.

### Primary and secondary outcomes

**Figure 2:**
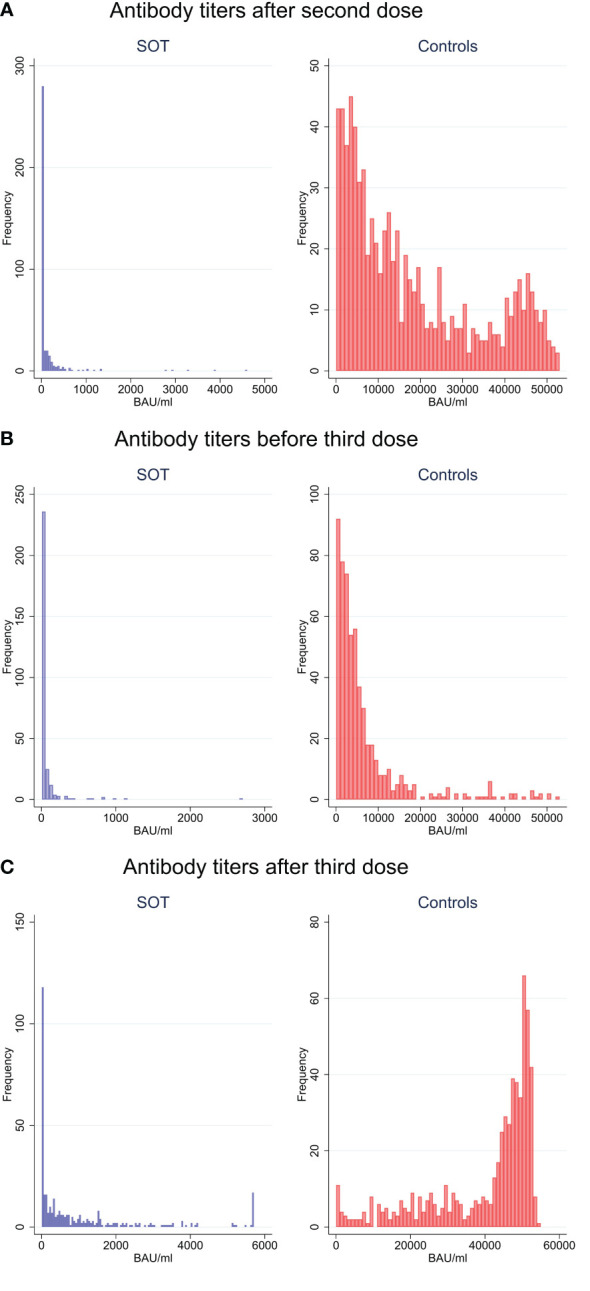
Anti-SARS-CoV-2 antibody concentrations to SARS-CoV-2 vaccine in solid organ transplant recipients and matched controls by frequency after second vaccine dose **(A)**, before third dose **(B)** and after third dose **(C)**.

**Figure 3:**
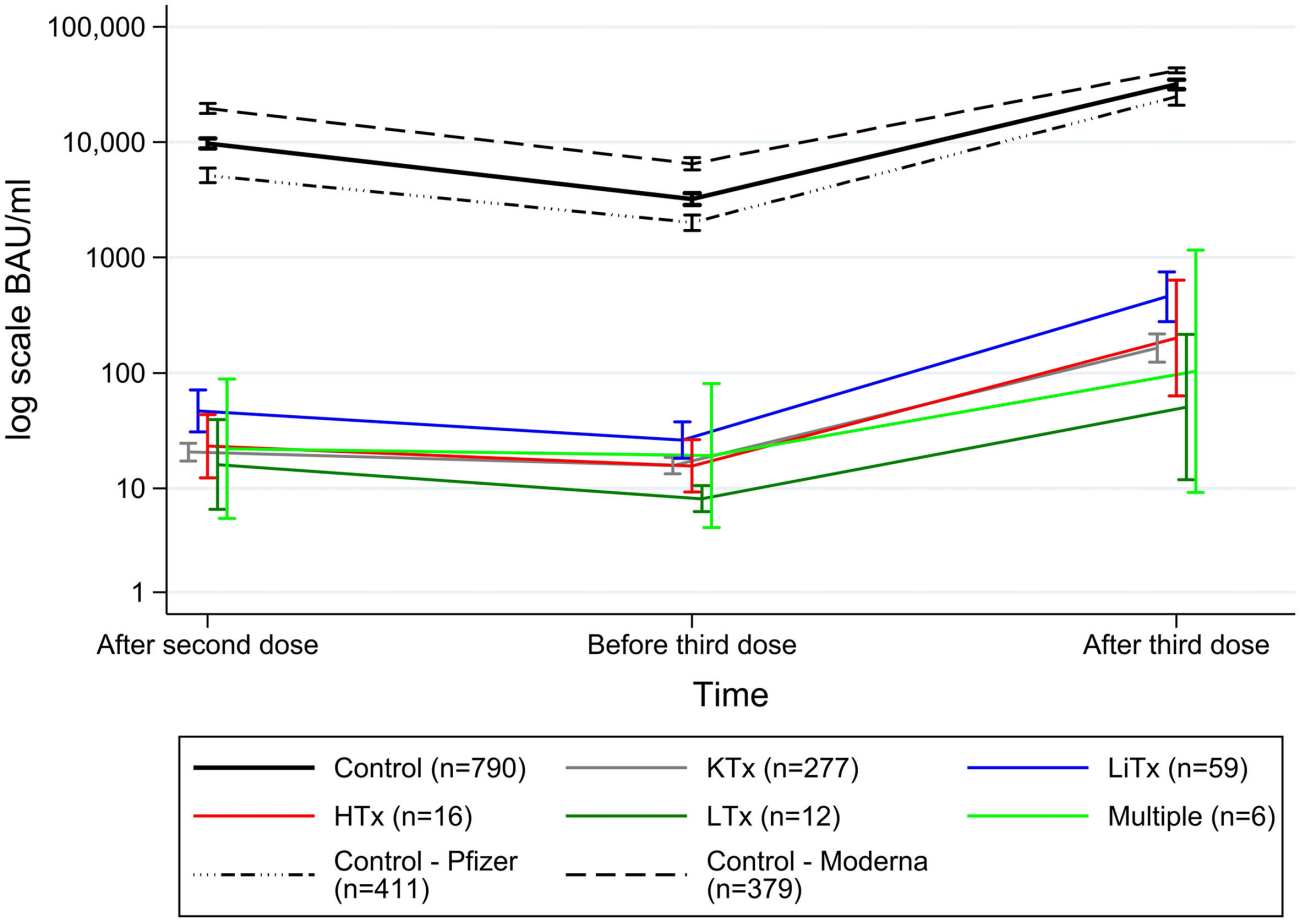
Mean (95%CI) anti-SARS-CoV-2 antibody concentrations to SARS-CoV-2 vaccine in solid organ transplant recipients and matched controls.

Serum antibody concentrations of the cases and controls following second and third vaccination are shown in **Table 2**, **Figures 2**, **3**. In total, SARS-CoV-2 spike S1 IgG antibodies were detected in 186/382 (49%) cases and in 781/790 (99%) controls after the second dose. Following the third dose, antibodies were detected in 275/358 (77%) cases and 601/609 (99%) controls. Overall, mean increase in antibody concentrations after the third dose was 893.3 BAU/ml (95%CI 740.3;1046.3) for SOT recipients and 22,114 BAU/ml (95%CI 20854;23373) for controls. Furthermore, the median antibody concentration after third dose for cases and controls was 332.6 BAU/ml (IQR 11.9-1349.4) and 46,470 BAU/ml (IQR 31,694-50,374), respectively. Among the 182 SOT recipients who were seronegative after the second dose, 100 (55%) became seropositive after the third dose, while one of the seropositive SOT recipients waned immunity (**Table 2**, **Figure 2C**).

**Figure 3** shows anti-SARS-CoV-2 antibody titers to SARS-CoV-2 vaccine correlated to transplant and controls stratified by the two different mRNA vaccine used. The liver transplant recipients had the highest antibody titers and the lung transplant recipients had the lowest. From after second dose to before third dose mean antibodies decreased by -70.1 BAU/ml (95%CI -104.2-36.0) for cases and by -10,223 BAU/ml (95%CI -11164;-9282) for controls. The controls who received mRNA-1273 (Moderna) had higher IgG Spike antibodies compared to those received BNT162b2 (Pfizer).

### Association of host-related factors and vaccinations response

Considering the impact of comorbidities on antibody concentrations in the SOT recipients, liver disease excluding liver transplant recipients and diabetes mellitus were associated with the lowest median antibody levels of 10.3 BAU/ml (IQR = 7.1-319.2) and 275.3 BAU/ml (IQR 7.3-957.4), respectively. Among controls, the same comorbidities were seen with median antibody concentrations of 43,422.2 BAU/ml (IQR 9128.1-50,907.3) and 44,835.0 BAU/ml (IQR 31,733.8-50,622.7) for liver disease and diabetes, respectively. The lowest median antibody concentration among controls was among cancer patients (39,709.6 BAU/ml IQR 17,122.2- 48,341.4).

Characteristics of breakthrough infections are shown in **Table 3**. Overall breakthrough infections were similarly frequent in both groups with 150 (40%) in the case and 301 (39%) in the control group (p = 0.80). Breakthrough infections were significantly more frequent among control participants after the second dose (p=0.03). When followed from January 1, 2022 through March 31, 2022, breakthrough infections were 35.25% vs 36.93% (p = 0.585) among cases and controls, respectively. The type of COVID-19 breakthrough variant was also comparable between groups, with Omicron being the most dominant type in both groups (89% in both groups). Thirteen patients were hospitalized due to COVID-19 and none of the cases died in the study period. The corresponding data for the controls were lacking.

**Table 3:** Characteristics of breakthrough infections among solid organ transplant recipients and matched controls.

Breakthrough infection	SOT recipients (n=377)Median (IQR) or n (%)	Controls (n=773)Median (IQR) or n (%)	*p*-value
**Total infections**	150 (40)	301 (39)	0.80
**Timing of infection**			
After first dose	0/377 (0)	1/773 (0.1)	>0.99
After second dose	3/377 (0.8)	22/773 (2.9)	0.03
After third dose	147/377 (39)	278/773 (36)	0.33
**Antibody concentration before infection**	123.5 (7.1-1059.7)	23336.9 (4960.7- 46567.1)	<0.001
**Variant**			>0.99
Delta	7 (4.6)	16 (5.3)	
Omicron	134 (89)	267 (89)	
Unknown	9 (6)	18 (6)	
**Characteristics**			
Hospitalization	13 (8.7)	Lacking data	
mAb treated	58 (39)	Lacking data	
Death	0 (0)	Lacking data	

Variants were decided on dates concerning outbreak waves in Denmark.

Delta:1.6.2021-1.12.2021, Omicron: 21.12.2021-1.4.2022, Unknown: Outside given intervals.

mAb, monoclonal antibodies.

## Discussion

This case-control study investigated differences in humoral response between SOT recipients and controls following a third dose of an mRNA-based SARS-CoV-2 vaccine. Our primary finding was that third dose had higher impact on SOT recipients due to an increasing proportion of responders compared to the sex- and age-matched control group. However, SOT recipients were still less likely to mount a positive antibody response after third dose (77% vs. 99% responders, p = <0.001) and had significantly lower antibody concentrations. Comorbidities as liver disease and diabetes mellitus were negatively associated with an antibody response in SOT recipients. Breakthrough infections were equally prevalent among cases and controls (40% vs 39%).

Our results on the humoral immunogenicity in SOT recipients parallel other studies. In our study, 77% of SOT recipients responded to a third dose which lies in the range of 49-80% as reported in other studies (6–10). In terms of seroconversion, our study showed that 55% of the seronegative SOT recipients became seropositive after the third dose, which approximately resembles the findings of 45% in the study by Del Bello et al (6). The findings of attenuated responses to COVID-19 have led several countries to recommend a fourth or even a fifth dose to SOT recipients, which has slightly improved humoral antibody response among patients with diminished response to a third dose (20, 21). In the present study, we observed a more pronounced decline in mean antibody concentrations from after second dose to before third dose in the control group compared to the cases (-10,223.0 BAU/ml vs -70.1 BAU/ml), despite the interval between second and third dose was significantly shorter for the control group (185 days vs 229 days, p <0.001). The immediate explanation to this could be the significant difference in antibody concentrations following second dose between the two groups with a higher starting point for the controls (7.1 BAU/ml vs 12,742 BAU/ml, p <0.001). After second dose, the SOT recipients antibody concentrations were close to zero meaning that SOT’s antibody response could not decline in the same degree as demonstrated in the control group.

Liver transplant recipients generally had better antibody response compared to other SOT recipients (14). A possible explanation is this group’s immunosuppressive protocol which contains a usual lower immunosuppressive treatment approach (two immunosuppressants) compared to other SOT groups (up to three immunosuppressant) and also that liver transplants are being generally well tolerated by the body (22, 23). Numerous studies have shown a similar association between high immunosuppression and low antibody response. In the study of Grupper et al., a 40% reduction in antibody response among kidney transplant recipients on triple vs. double immunosuppressive treatment was found (4). Therefore, a lower degree of immunosuppression seems to facilitate higher antibody responses as observed among liver SOT vs. other SOT types.

We were unable to compare rate of admission and mortality between groups in relation to breakthrough infection. In the ENFORCE cohort, quantitative level of spike antibody had limited impact on the risk of breakthrough infection with Omicron compared to Delta variant and the hospitalization rate was 0.2% (24). In a systematic review and meta-analysis by Gatti et al., they found no increased risk in mortality in SOT recipients with COVID-19 compared to the general population when adjusted for demographics and clinical features such as comorbidities and COVID-19 severity (25). Another meta-analysis by Raja MA et al. concluded that the overall outcome among SOT recipients was equal to the general population, however, the admission rate was found to be higher (26).

In Denmark, SOT recipients were a highly prioritized group receiving vaccination at earlier time points than the general population, thus the timing of vaccinations and in-between-intervals for cases differs from that of the controls (**Figure 1**). This difference may influence the prevalence of breakthrough infections following the third dose as SOT recipients are followed for longer time periods, thus being more exposed to breakthrough infections. Therefore, we performed an additional analysis where cases and controls with no prior infection were followed from January 1, 2022 through March 31, 2022. Doing this, we obtained a similar background risk of breakthrough infections between groups and still found no significant differences 35.25% vs 36.93% (p = 0.585), cases and controls, respectively.

Limitations of this study include a lack of standardized assay used for antibody measurement throughout the study period for cases and controls. We were still able to compare antibody concentrations by converting the Mesoscale arbitrary unit AU/ml to the BAU/ml unit, which was used for cases. Another limitation of this study was missing antibody values for both cases and controls after the third dose, which may have underestimated the proportion of responders and the antibody concentrations. Furthermore, there was a difference in vaccines used in controls compared to the cases. Among controls 52% were Pfizer vaccinated and 48% Moderna vaccinated while 100% of cases were Pfizer vaccinated. In the general population both vaccines has showed comparable efficacy of 95% (Pfizer) and 94.5% (Moderna) (27). In contrast to this, in the ENFORCE study, vaccine type was an independent risk factor for hyporesponsiveness (Moderna compared to Pfizer adjusted OR= 0.14, 95% CI: 0.09-0.22) (28). Furthermore, the controls who received the Moderna vaccine had higher Spike IgG titers. In addition, we did not profile the cellular immunity following SARS-CoV-2 vaccination. Even though the cellular response was shown to be low among SOT recipients compared to healthy controls (29), and no T-cell immunity threshold is defined, we would expect additional immune response elicited in both SOT recipients and controls (Data in review). Lastly, we were unable to provide data regarding immunosuppressive treatment, some characteristics of breakthrough infections and hospitalization among the control participants.

## Conclusion

In SOT recipients, the third dose of COVID-19 vaccination increased the humoral response and the proportion of responders. However, overall antibody concentrations were still considerably lower compared to controls. In SOT recipients, humoral immunity was highly influenced by the presence of liver disease and diabetes, whereas breakthrough infections occurred with equal prevalence in both groups.

## Data availability statement

The raw data supporting the conclusions of this article will be made available by the authors, without undue reservation.

## Ethics statement

The COVAC-Tx study was conducted in accordance with the Declaration of Helsinki and approved by the Regional Committees on Health Research Ethics for Southern Denmark January 29, 2021 (protocol code S-20210007C) and the amendment approved February 9, 2022. The ENFORCE study was approved by the Danish Medicines Agency (Eudra CT number:2020-006003-42) and by the Ethics Committee of the Central Denmark Region (#1-10-72-337-20). The patients/participants provided their written informed consent to participate in this study.

## Author contributions

IJ, UJ, AN, CB, RA, JD, and MP were responsible for conception and design. CB, RA, JD, and MP and DB collected data. AN was responsible for laboratory data. IJ and SL are responsible for project administration. IP was responsible for the analyses. DB and IJ were responsible for drafting the manuscript. All the authors were responsible for reviewing and revising the manuscript. All authors contributed to the article and approved the submitted version.
