# Supplementary material for: Humoral immune response following a third SARS-CoV-2 mRNA vaccine dose in solid organ transplant recipients compared with matched controls
Source: Front Immunol. 2022 Dec 9;13:1039245. doi: 10.3389/fimmu.2022.1039245 (PMC9780530; doi:10.3389/fimmu.2022.1039245)
Supplement: Supplementary file 1 [file DataSheet_1.pdf]

ENFORCE

Danish National Cohort Study of Effectiveness and Safety of SARS-CoV-2 vaccines

ENFORCE STUDY GROUP

midt regionmidtjylland

REGION H Rigshospitalet

Sponsor/Data and Statistical Centre: CHIP, Rigshospitalet under the direction of Professor J Lundgren.

Principal Investigator: Professor LJ Østergaard, Department of Infectious Diseases, Aarhus University and Aarhus University Hospital.

Sites (alphabetical and regional coordinators in parenthesis)

Region Hovedstaden: (T Benfield), L Krohn-Dehli, DK Petersen, Copenhagen University Hospital - Amager and Hvidovre, Hvidovre. K Fogh, EH Mikkelsen, (K Iversen), Copenhagen University Hospital – Gentofte and Herlev, Herlev.

Region Midtjylland: P Bek, V Klastrup, F Larsen, SH Rasmussen, MH Schleimann, S Schieber, (NB Stærke), A Søndergaard, B Tarp, M Tousgaard, Y Yehdego, Aarhus University Hospital, Aarhus.

Region Nordjylland: J Bodilsen, (H Nielsen), KT Petersen, M Ruwald, RK Thisted, Aalborg University Hospital, Aalborg.

Region Sjælland: SF Caspersen, M Iversen, LS Knudsen, JL Meyerhoff, LG Sander, (L Wiese), Zealand University Hospital Roskilde, Roskilde.

Region Syddanmark: C Abildgaard, IK Holden, NE Johansen (IS Johansen), L Larsen, SO Lindvig, LW Madsen, A Øvrehus, Odense University Hospital, Odense.

Scientific Steering Committee: NA Kruse, H Lomholdt, Lægemiddelstyrelsen, TG Krause, P Valentiner-Branth, Statens Seruminstitut, B Søborg, Sundhedsstyrelsen, TK Fischer, Copenhagen University, C Erikstrup, Aarhus University, SR Ostrowski, Rigshospitalet, H Nielsen, Aalborg University Hospital, IS Johansen, Odense University Hospital, LJ Østergaard (chair), M Tolstrup, NB Stærke, OS Søgaard Aarhus University Hospital, L Wiese, Zealand University Hospital Roskilde, T Benfield, Copenhagen University Hospital – Amager and Hvidovre, J Lundgren, D Raben, CHIP, Rigshospitalet

Operational group: H Nielsen, Aalborg University Hospital, IS Johansen, Odense University Hospital, LJ Østergaard, M Tolstrup, NB Stærke, OS Søgaard, Aarhus University Hospital, L Wiese, Zealand University Hospital Roskilde, T Benfield, Copenhagen University Hospital – Amager and Hvidovre, J Lundgren (chair), D Raben, CHIP, Rigshospitalet, E Jylling, Danske Regioner, D Hougaard, Statens Seruminstitut

Coordinating Centre: Aarhus University and Aarhus University Hospital, SD Andersen, K Lykkegaard, NB Stærke, OS Søgaard, M Tolstrup, LJ Østergaard

ENFORCE Lab.: Aarhus University Hospital: SR Andreasen, E Baerends, LL Dietz, AK Hvidt, AK Juhl, R Olesen, M Tolstrup.

Data and Statistical Centre: CHIP, Rigshospitalet: KK Andersen, W Bannister, C Bjernved, TW Elsing, FV Esmann, MA Ghafari, E Gravholdt, SF Jakobsen, ML Jakobsen, CM Jensen, TØ Jensen, D Kristensen, LR Kumar, J Lundgren, C Matthews, N Normand, C Olsson, D Raben, J Reekie, A Traytel, T Weide.

Other contributors: AM Hvas, H Støvring, Aarhus University Hospital, Statens Seruminstitut

Funding: ENFORCE has received a grant from the Danish Ministry of Health (SUM) (legal deeds 150 28/1 2021 and 263 3/6 2021)
